# Atomic-scale structure and properties of highly stable antiphase boundary defects in Fe_3_O_4_

**DOI:** 10.1038/ncomms6740

**Published:** 2014-12-10

**Authors:** Keith P. McKenna, Florian Hofer, Daniel Gilks, Vlado K. Lazarov, Chunlin Chen, Zhongchang Wang, Yuichi Ikuhara

**Affiliations:** 1 Department of Physics, University of York, Heslington, York YO10 5DD, UK; 2 WPI-AIMR, Tohoku University, 2-1-1 Katahira, Aoba-ku, Sendai 980-8577, Japan

## Abstract

The complex and intriguing properties of the ferrimagnetic half metal magnetite (Fe_3_O_4_) are of continuing fundamental interest as well as being important for practical applications in spintronics, magnetism, catalysis and medicine. There is considerable speculation concerning the role of the ubiquitous antiphase boundary (APB) defects in magnetite, however, direct information on their structure and properties has remained challenging to obtain. Here we combine predictive first principles modelling with high-resolution transmission electron microscopy to unambiguously determine the three-dimensional structure of APBs in magnetite. We demonstrate that APB defects on the {110} planes are unusually stable and induce antiferromagnetic coupling between adjacent domains providing an explanation for the magnetoresistance and reduced spin polarization often observed. We also demonstrate how the high stability of the {110} APB defects is connected to the existence of a metastable bulk phase of Fe_3_O_4_, which could be stabilized by strain in films or nanostructures.

## Abstract (web summary)

Although Fe_3_O_4_ is widely investigated for a variety of applications, the relation between some defects and its properties remains poorly understood. Here, the authors use high-resolution transmission electron microscopy and simulations to determine the atomic structure of the common antiphase boundary defects.

## Graphical abstract

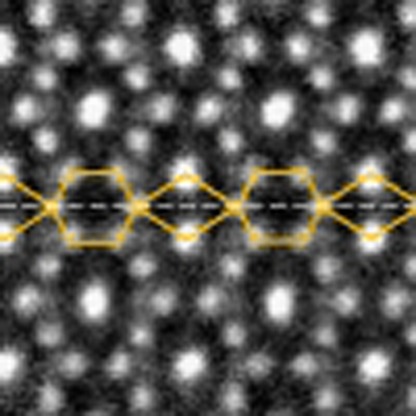

Magnetite (Fe_3_O_4_) is one of the most abundant iron-containing minerals on our planet and the oldest known magnetic material. It finds diverse applications in areas such as catalysis, rechargeable batteries, magnetic recording, medicine and biology^1^,^2^,^3^,^4^,^5^,^6^,^7^ and continues to receive fundamental interest owing to its complex and intriguing electronic properties^8^,^9^,^10^. At room temperature, magnetite is predicted to be a half-metallic ferromagnet (a material whose conduction electrons are 100% spin polarized), making it attractive for spintronic applications such as magnetic memories and spin field-effect transistors^3^,^4^,^5^. However, the experimentally measured spin polarization (whether determined spectroscopically or through transport measurements) is always much lower^11^,^12^. One of the primary culprits thought to reduce spin polarization are antiphase boundary (APB) defects that are common in magnetite films and bulk polycrystals. It is suggested that the perturbation in atomic structure at such defects may modify superexchange interactions between the magnetic moment-carrying Fe atoms either side of APBs leading to antiferromagnetic (AF) coupling between adjacent structural domains^13^,^14^. While the presence of APB defects in Fe_3_O_4_ is well known (for example, from electron or scanning probe microscopy studies^15^,^16^) precise determination of their atomic-scale structure and magnetic properties has proved challenging. As a result, the presence of AF superexchange interactions in APBs has largely been inferred from crystallographic arguments rather than from direct experimental or theoretical evidence^17^,^18^. While these simple models are often very useful, they bring no information on the relative stability or electronic properties of APB defects, which presents a significant obstacle to developing a deeper understanding of their role to optimize materials for applications.

At room temperature, bulk magnetite is a ferrimagnetic inverse spinel (space group 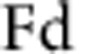) with Fe ions occupying both tetrahedral (tet) and octahedral (oct) sites with oxidation states 3+ and 2.5+, respectively. Below 120 K, the electrons on the octahedral sites form a polaronic charge-ordered state inducing a monoclinic distortion (known as the Verwey transition)^8^,^9^,^10^,^19^. Although at room temperature bulk Fe_3_O_4_ is predicted to have 100% spin polarization at the Fermi level^3^,^20^, it has proven challenging to verify this experimentally owing to uncertainties associated with material stoichiometry, the role of surface reconstructions and the presence of various defects^11^,^12^,^21^. Even the highest quality epitaxial films contain numerous APB defects that form during growth due to island coalescence^22^,^23^. These APB defects have been invoked to explain the large negative magnetoresistance observed in Fe_3_O_4_ (ref. 13). In line with the Goodenough–Kanamori rules^24^,^25^, if APBs introduce Fe–O–Fe bond angles close to 180°, they will give rise to superexchange interactions coupling the adjacent domains antiferromagnetically^26^,^27^. Therefore, in the absence of a magnetic field, the resistance will be high due to strong scattering of the spin-polarized electrons at APBs^22^. On application of a field, the alignment between magnetic moments in different domains can be improved, reducing electron scattering and giving rise to a decrease in resistance^13^. However, direct information concerning the atomic structure and magnetic properties of APBs is currently lacking. Several atomic models of APBs have been built on the basis of crystallographic arguments^17^,^18^,^28^. For example, APBs on the {110} planes have been suggested to involve (1/4)*a*‹110› and (1/2)*a*‹100› crystal translations. In one case, this type of model has been used as the basis for first principles electronic structure calculations^29^. However, there has been no systematic search for stable APB configurations at a theoretical level and no experimental determination of the atomic structure of APBs limiting our ability to correlate the structure and properties of these important defects.

In this article, we employ first principles-based theoretical modelling to predict the detailed atomic structure, magnetic and electronic properties of stable APB defects in magnetite. Our approach is applied to consider APBs forming on the {110} planes—a common type of APB seen in Fe_3_O_4_ materials. Following a thorough theoretical screening of structures, we identify two possible {110} APB structures both of which are predicted to couple adjacent domains antiferromagnetically. Importantly, we show that the most stable APB structure predicted theoretically is in excellent agreement with high-resolution transmission electron microscopy (TEM) analysis confirming the validity of our predictive approach. This APB has an extremely low formation energy that can be rationalized as it locally resembles a metastable Fe_3_O_4_ phase (space group Pmma), which is only 332 meV per formula unit less stable than the room temperature cubic phase. The metastable Pmma phase has to the best of our knowledge never been observed as a bulk phase, but could in principle be stabilized by strain in nanostructures or formed in a non-equilibrium growth process^30^.

## Results

### Prediction of stable {110} APB defects

**Figure 1:**
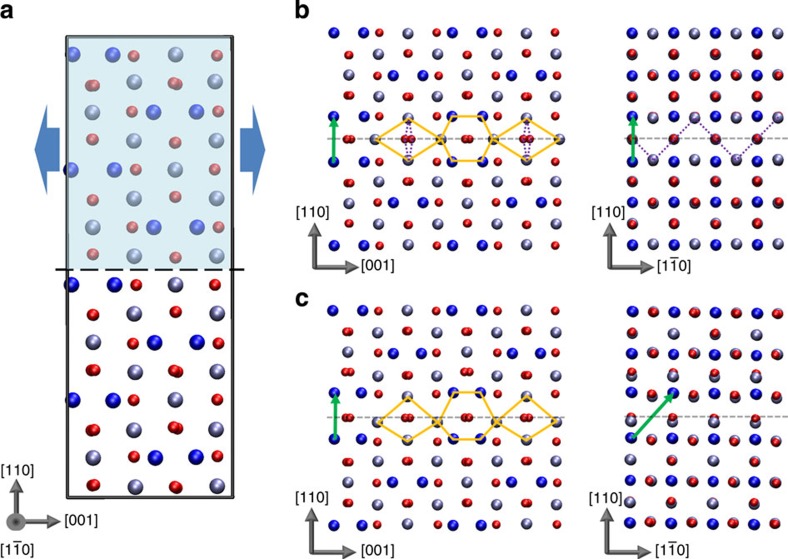
Predicted {110} APB defects in magnetite. (**a**) The ideal cubic Fe_3_O_4_ structure showing the approach employed to search for stable APB structures. (**b**) APB-I characterized by crystal translation (1/4)*a*[110]. (**c**) APB-II characterized by crystal translation 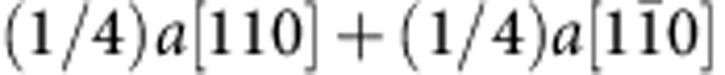. The APB crystal translations are indicated by green vectors in the figures. Small red spheres represent oxygen atoms, large dark blue spheres represent tetrahedral Fe atoms and light blue spheres represent octahedral Fe atoms. Orange diamonds and hexagons highlight the structural units and broken purple lines highlight the 168° Fe–O–Fe bonds present at the interface.

Our theoretical approach to predict the structure and properties of APB defects in Fe_3_O_4_ follows that which we have employed previously to model extended defects in a range of metal-oxide materials^31^,^32^,^33^. Briefly, our hierarchical approach begins with a systematic screening of possible APB configurations using classical interatomic potentials to describe the interactions between ions. Here we employ an ionic potential similar to that used previously for modelling defect formation energies and vibrational properties of Fe_3_O_4_ (refs 34, 35). Calculations are performed using three-dimensional (3D) periodic supercells containing two equivalent {110} APBs with eight atomic planes in each crystal (thickness of ~12 Å). We search overall possible relative crystal translations using a grid spacing of 0.1 Å (see arrows in Fig. 1a). Following this search, only a small number of inequivalent stable configurations are obtained, which are fully optimized at the density functional theory (DFT) and DFT+ *U* levels. APB formation energies are then calculated, which represent the stability of the defect with respect to the perfect bulk crystal (see Methods). On thermodynamic grounds, one usually expects that APBs with the lowest formation energy will be more common; however, in non-equilibrium growth processes, kinetic factors may also be important making it less straightforward to predict which APB defect is most likely to appear. In the following, all energies are given first for the DFT+ *U* method, with the DFT values given in parentheses.

Following the theoretical search for possible {110} APB structures in magnetite, we find only two stable configurations, which are shown in Fig. 1 (see Supplementary Data Sets 1–4 for coordinates of the optimized structures). The most stable APB (denoted APB-I) is characterized by a crystal translation (1/4)*a*[110] and its formation energy is very low, only 102 mJ m^−2^ (307 mJ m^−2^). The low formation energy is consistent with the fact that introduction of the APB leads to no net change in Fe coordination (that is, the number of octahedral and tetrahedral Fe sites is the same as in the bulk crystal). Only the ordering of octahedral and tetrahedral Fe sites between the (111) oxygen planes is modified leading to a relatively small structural distortion near the interface (see Supplementary Figs 1 and 2; Supplementary Discussion). We note that such a low formation energy for an extended defect in an oxide is highly unusual and we return to this point later in the manuscript. The other stable APB (denoted APB-II) is characterized by a crystal translation 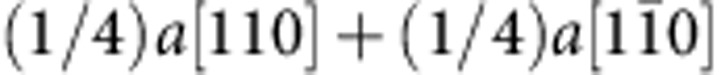 and has a larger formation energy of 954 mJ m^−2^ (967 mJ m^−2^). Viewed along the 
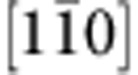
 direction both APB-I and APB-II appear similar, with the interfacial Fe sites defining repeating structural units of hexagons and diamonds. However, in APB-II, the structural units are asymmetric and distorted indicating a higher degree of structural perturbation consistent with the higher formation energy. The additional crystal translation in the 
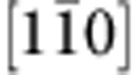
 direction leads to the introduction of Fe–Fe bonds that are about 0.5 Å shorter than in the bulk explaining the lower stability of APB-II and increased structural distortion. We have verified through calculation of vibrational frequencies at the classical potential level that APB-I and APB-II are locally stable (that is, all vibrational modes are real and there are no soft modes that could facilitate a transformation to the bulk structure). For both APBs, we find that the most stable magnetic configuration involves the two ferrimagnetic domains coupled antiferromagnetically to each other across the interface. We note that stable configurations involving pure crystal translations of (1/2)*a*[100] were not found consistent with the very low frequency of observation of these shifts in films^18^.

### TEM study of {110} APBs

**Figure 2:**
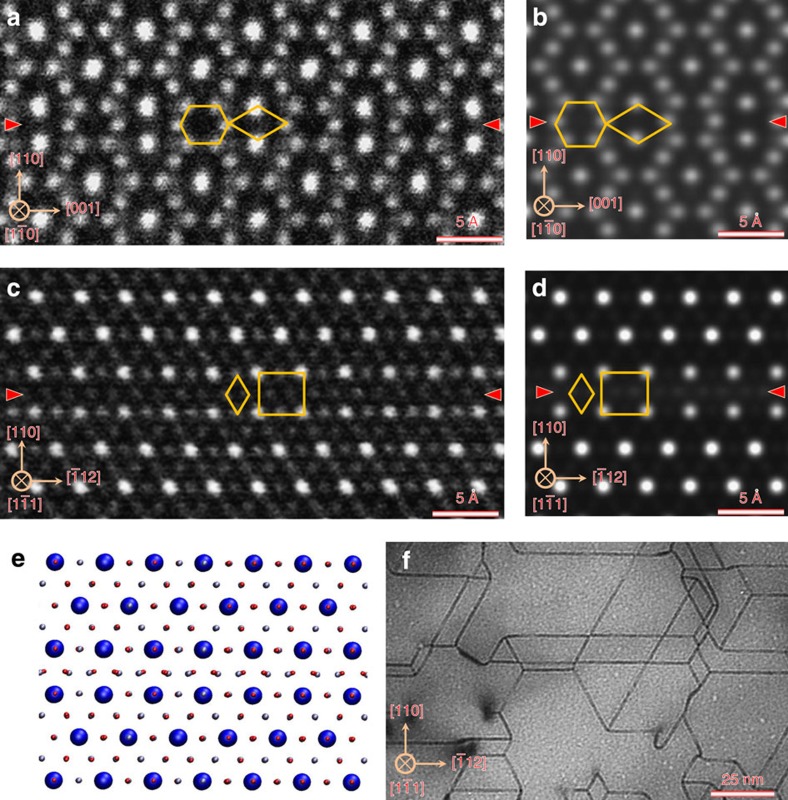
TEM of the {110} APB defects in magnetite. (**a**) HAADF-STEM image of the (110) APB along the 
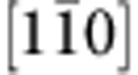
 zone axis. (**b**) Simulated HAADF-STEM image for the theoretically predicted structure APB-I along the 
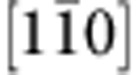
 zone axis. (**c**) HAADF-STEM image of the (110) APB along the 
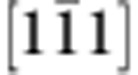
 zone axis. (**d**) Simulated HAADF-STEM image for the theoretically predicted structure APB-I along the 
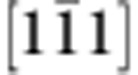
 zone axis. (**e**) Atomic model for APB-I along the 
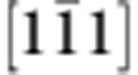
 zone axis. (**f**) Bright-field TEM image of the {110} APB network along the 
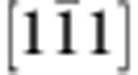
 zone axis obtained after annealing process. The red arrows indicate the APBs.

To verify whether the theoretically predicted APB structure agrees with that seen in real materials, we have performed a TEM study of APBs in Fe_3_O_4_. The Fe_3_O_4_ films employed were produced by thermally annealing thin films of Fe_2_O_3_ with thicknesses ranging from several nanometres to tens of nanometres under a pressure of 10^−4^ Pa at 973 K for 1 h. Following this procedure, high-quality Fe_3_O_4_ films containing a number of {110} APBs were obtained. To resolve the APB structure with sub-Ångström resolution, high-angle annular dark-field (HAADF) images were taken with a 200-kV scanning TEM (STEM). Figure 2a shows a HAADF-STEM image of the (110) APB along the 
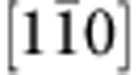
 zone axis. To allow direct comparison with the theoretical models, a HAADF-STEM image simulation has been performed using the coordinates obtained from the DFT calculation on APB-I using the multi-slice method^36^ (Fig. 2b). The agreement between the experimental and simulated image is remarkable—clearly showing the hexagon and diamond structural units. The high symmetry, particularly in the hexagonal structural unit, provides clear discrimination between the APB-I and APB-II models consistent with the lower formation energy of the former. The atomic structure of (110) APB along the 
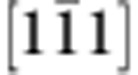
 zone axis has been also investigated by the HAADF-STEM technique, as shown in Fig. 2c, providing 3D information on its structure. This HAADF-STEM image also exhibits very good agreement with the simulated HAADF image of the APB-I structure as shown in Fig. 2d, which is obtained according to the atomic model shown in Fig. 2e. The excellent agreement again confirms that the {110} APBs formed during annealing process correspond to APB-I. The fact that for this sample the most stable {110} APB defect is realized suggests that the APB formation is thermodynamically driven as one may expect given that it has been formed through thermal annealing. The density of the {110} APBs formed during the annealing process can be seen from the bright-field TEM image taken along the 
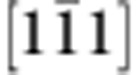
 zone axis (Fig. 2f). In this image, we can see {110} APBs with a density of about 5 × 10^15^ m^−2^ forming a network structure. These {110} APBs can be indexed as (110), (011) and 
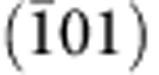
 APBs and have intersection angles of 60°. We also performed HAADF-STEM analysis on magnetite films grown on single-crystal MgAl_2_O_4_(111) substrates by molecular beam epitaxy methods and find similar agreement with the APB structure (see Supplementary Fig. 3; Supplementary Discussion). However, there appears to be fewer {110} APBs present in the film sample suggesting that non-equilibrium growth process may play a more important role.

### Electronic and magnetic properties

**Figure 3:**
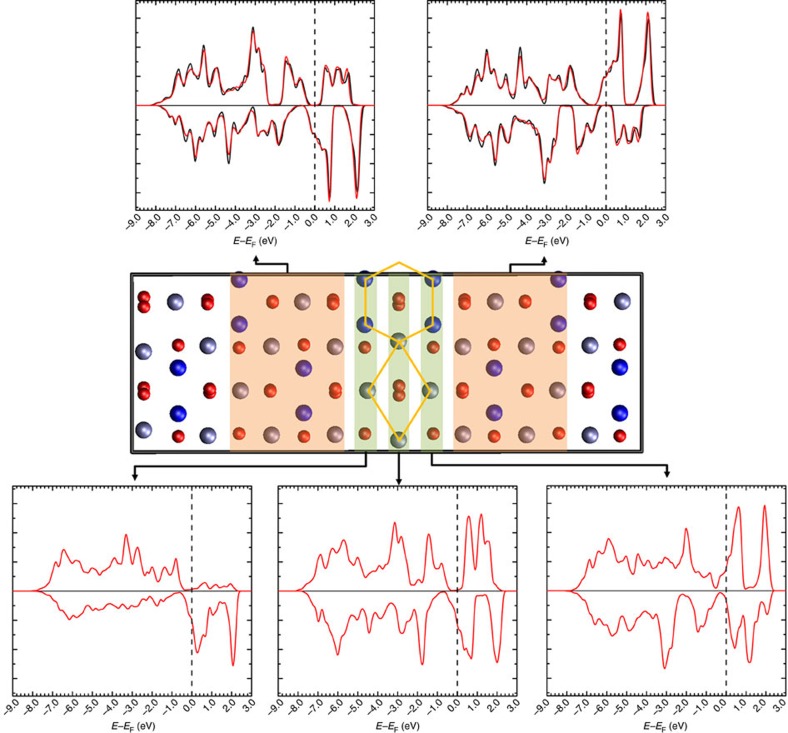
The spin-polarized DOS of the {110} APB defect in Fe_3_O_4_. The DOS (red lines) is projected onto regions in the vicinity of the APB indicated by the shaded areas. In the bulk-like regions either side of the APB (top), the DOS is almost indistinguishable from the DOS of the bulk crystal (black lines).

Experimentally, isolating the magnetic properties of individual APBs in magnetite is extremely challenging, but with the atomic-scale structure determined precisely we can calculate its properties directly using DFT. In particular, as noted above, the predicted magnetic configuration involves the two ferrimagnetic domains coupled antiferromagnetically. We have also computed the energy required to align the magnetization of both domains in APB-I with the position of ions held fixed, providing a measure of the strength of the AF coupling. We find that it costs 49 mJ m^−2^ (16 mJ m^−2^) to overcome the exchange interactions across the APB. Analysis of the atomic structure near the interface indicates the presence of Fe(oct)–O–Fe(oct) bonds with an angle of 168°, which are responsible for the AF coupling though the superexchange mechanism (highlighted in Fig. 1). Turning to the electronic properties, Fig. 3 shows the spin-polarized density of states (DOS) of a {110} APB defect Fe_3_O_4_ showing that there is a gradual transition from positive to negative spin polarization across the three atomic layers near the APB defect. We present the DOS calculated at the DFT level rather than the DFT+ *U* level since it provides a better representation of the half-metallic electron structure above the Verwey transition, which is most relevant for spintronic devices and electron transport spin polarization measurements. At the DFT+ *U* level, insulating charge-ordered electronic ground states are obtained consistent with the low transition temperature for the Verwey transition and previous calculations^37^. In the ground state AF configuration, the spin polarization reverses direction across the APB plane, hence the transport of electrons between the domains in both spin states will be associated with substantial scattering. In the ferromagnetic configuration, the total DOS is fully polarized and very similar to the bulk DOS reflecting the fact that few interface states are introduced by the APB defect.

### Structure and properties of the Pmma phase of Fe_3_O_4_

**Figure 4:**
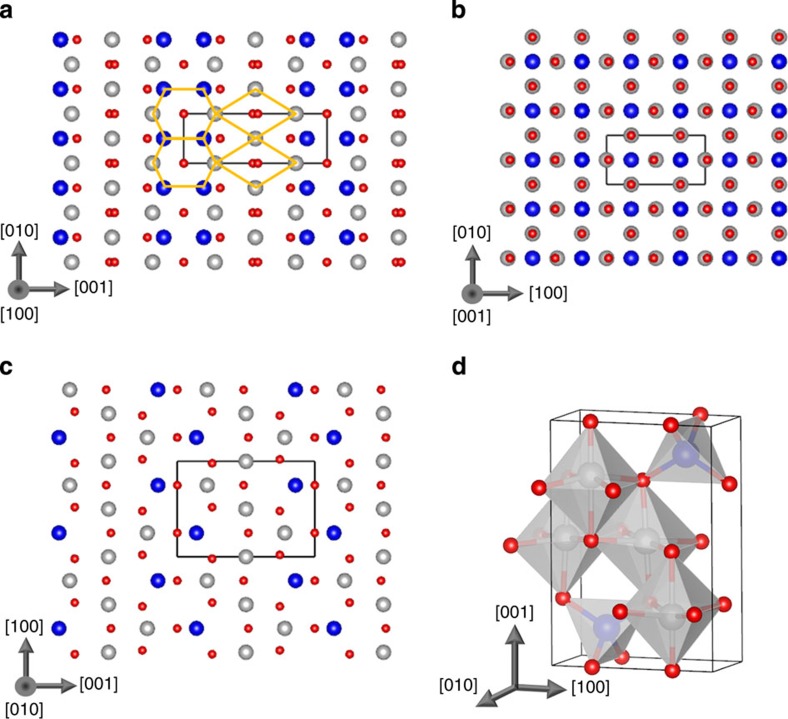
Predicted structure of the Pmma phase of Fe_3_O_4_. (**a**–**c**) Views of the unit cell along the principal crystallographic directions. Small red spheres represent oxygen atoms, large dark blue spheres represent tetrahedral Fe atoms and grey spheres represent octahedral Fe atoms. Orange diamonds and hexagons highlight the structural units. (**d**) A 3D view of the unit cell showing the Fe octahedra and tetrahedra. Figures produced using the VESTA package^47^.

We now return to the unusual finding that the formation energy of the APB-I defect is so low—only 102 mJ m^−2^. This suggests that thermodynamically many such APB defects may form during growth or annealing that would seriously affect the performance of devices. Typically, extended defects and surfaces in materials have formation energies of the order 1 Jm^−2^. Much lower formation energies can be found for stacking faults in some materials if there are alternative crystalline phases with similar stability. For example, both theoretical calculations and experimental measurements find that the {111} stacking fault energy in face-centred cubic (fcc) copper is of the order 40–70 mJ m^−2^ (ref. 38). The fcc stacking fault can be represented schematically in terms of the sequence of {111} planes of the form ABC|BCA (where | indicates the stacking fault plane). The low formation energy of this defect can be understood because the stacking BCBCBC corresponds to the bulk hexagonal close-packed phase, which is only a few meV per atom less stable than the fcc structure. This raises an interesting question as to whether the low formation energy of the APB-I defect can be a result of the local stacking resembling a metastable bulk phase. For instance, one can construct an orthorhombic crystal structure consisting of a periodic arrangement of the hexagon and diamond structural units found in APB-I. Optimizing this structure at the DFT+ *U* level, one finds a crystal with space group Pmma and lattice parameters: *a*=3.086 Å, *b*=8.654 Å and *c*=5.898 Å (Fig. 4; also see Supplementary Data Sets 5 and 6 for coordinates of the optimized structures).

The Pmma phase is predicted to be only 332 meV per formula unit less stable than the cubic 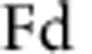 phase. There are similarities with the cubic phase as it also consists of an arrangement of Fe-centered octahedra and tetrahedra; however, in the Pmma phase, two of the octahedra in the unit cell are slightly distorted. The Pmma phase is much more stable than many other metastable phases of Fe_3_O_4_—for example, the Pbcm phase is a stable high-pressure phase but it is about 1 eV per formula unit less stable than the 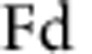 phase^39^. The unit cell volume of the Pmma phase is larger than 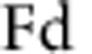 by 2.8 Å^3^ per formula unit, suggesting that this phase would not be stable as a bulk material under high pressure but could perhaps be stabilized in thin films by strain. We find that the ferrimagnetic configuration is favoured over the AF one with a magnetic moment of 3.9 *μ*_B_ per formula unit. At the DFT+ *U* level, the Pmma phase exhibits charge ordering over the octahedral Fe sites resulting in an electronically insulating state. Very similar properties are predicted at the DFT level with the exception that the electronic ground state is predicted to be metallic with a 35% spin polarization at the Fermi level (see Supplementary Fig. 4; Supplementary Discussion). The existence of this metastable phase has useful explanatory power for understanding the very low formation energy of the {110} APB. However, we note that there are some factors that are important for stabilizing a bulk crystal, which will not necessarily be important at an interface (such as Madelung potential and lattice expansion/contraction). As far as we are aware, the Pmma phase has never been observed but could in principle be stabilized by strain in nanostructures or grown in a non-equilibrium process^30^.

## Discussion

In summary, we have predicted the atomic structure of {110} APB defects in magnetite and find that they are characterized by an extremely low formation energy. The predicted atomic structure of the most stable APB defect has subsequently been verified in all three dimensions by high-resolution electron microscopy highlighting the predictive power of our theoretical approach. Determination of the APB defect structure with such unprecedented atomistic detail has opened the door to prediction of its corresponding electronic and magnetic properties. We note that the properties predicted at the DFT and DFT+ *U* levels are largely consistent with the latter exhibiting charge ordering as expected below the Verwey transition temperature. We have shown that the most stable {110} APB induces AF coupling between adjacent ferrimagnetic domains and have calculated from first principles the energy required to align the magnetization of adjacent domains separated by an APB (49 mJ m^−2^ at the DFT+ *U* level). This provides much needed input into first principles-based micromagnetic simulations of magnetization dynamics in magnetite with relevance for applications in magnetic hyperthermia and spintronic device modelling. We also show how the unusually low formation energy of the APB can be understood in terms of its similarity to a metastable magnetite bulk phase. Although this phase is not predicted to be stable under atmospheric or high pressure, it could be formed in films or nanoparticles as a result of strain or finite-size effects. It is possible that ultrastable APBs may be present in a broader class of similar spinel materials, which are of fundamental and technological significance. Aside from this practical relevance, there has been a great deal of interest in low-temperature charge ordering (Verwey transition) both in the bulk^8^,^9^,^10^ and at surfaces^40^ of Fe_3_O_4_. Studying the influence of 2D APB defects on charge ordering may reveal yet more exotic effects helping to deepen our understanding of these correlated electron systems. The combination of predictive first principles theoretical calculations and high-resolution TEM presented here provides invaluable insight into the atomic structure of the ubiquitous APB defects in Fe_3_O_4_ that affect the performance of materials for wide-ranging applications in spintronics, catalysis and medicine.

## Methods

### Determination of stable APB structures

We employ a two-stage modelling approach for predicting the stable structures of APB defects in Fe_3_O_4_ as previously used to model extended defects in other oxides such as MgO, TiO_2_ and HfO_2_ (refs 31, 32, 33, 41). At the first level of modelling, we describe the interactions between Fe and O ions with a classical pair potential approach similar to that employed in other studies of the surface and defect properties of Fe_3_O_4_ (refs 1, 34). Interactions between the ions are described with the following Buckingham potential functional form:


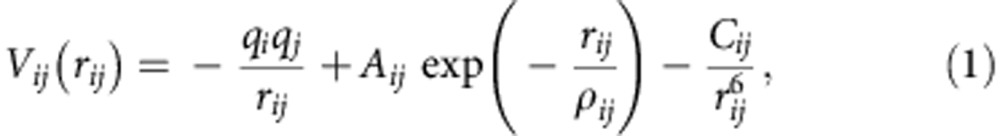


where *r*_*ij*_ is the distance between a pair of ions labelled with indices *i* and *j*, *q* is the corresponding ion charge and *A*, *ρ* and *C* are species-dependent potential parameters. Most existing pair potential models of Fe_3_O_4_ consider oxygen ions with formal charge *q*_O_=−2, tetrahedrally (tet) coordinated Fe ions with formal charge *q*_tet_=+3 and the octahedrally coordinated Fe ions as a 50/50 mixture of sites with formal charge *q*_oct_=+2 and *q*_oct_=+3 (ref. 34). This models the trapping of electrons on one half of the octahedral Fe sites, which is expected below the Verwey transition. However, for extended defects, where it is not known *a priori* where the additional electrons will be located, such a description becomes problematic. Therefore, here we develop a simpler model in which tetrahedral Fe ions have a formal charge *q*_tet_=+3 and octahedral Fe ions ions have a formal charge *q*_oct_=+2.5 corresponding to delocalization of the excess electrons over all octahedral sites (appropriate above the Verwey transition). We also simplify the description by considering non-polarizable O and Fe ions. We take the pair potential parameters for O–O and Fe(tet)–O from a previous study that fitted them to give good agreement with the experimental phonon spectrum and crystal structure^35^. The cut-off for all short-range interactions is set to 12 Å. The *A* parameter in the Fe(oct)–O interaction is refitted to yield the experimental lattice constant of 8.397 Å and the full set of parameters obtained is shown in Table 1. We note that the fitted *A* parameter for the F(oct)–O interaction is between those previously fitted for +2 and +3 Fe in octahedral sites as expected^34^.

Using the classical pair potential approach described above, we model (110) APB defects within a periodic supercell as shown in Fig. 1a. Two equivalent APB defects separating two grains each consisting of eight atomic planes (thickness of ~12 Å) are introduced to maintain 3D periodicity. We initially translate one grain relative to the other in both directions parallel to the APB plane in steps of 0.1 Å (see arrows in Fig. 1a) before optimizing these structures with respect to the positions of all ions and the length of the supercell in the direction perpendicular to the APBs. Through this systematic searching approach, we are able to find all stable APB structures. These stable structures are then re-optimized using DFT to determine the formation energy, electronic and magnetic properties.

### Periodic DFT calculations

Spin-polarized (collinear) DFT calculations are performed using the projector augmented wave (PAW) method as implemented within the Vienna *ab initio* simulation package^42^,^43^. The standard PAW potential for Fe and the soft PAW potential for O are employed (we verified employing harder potentials or including semi-core states that had little effect on electronic or magnetic properties). We use the Perdew–Burke–Ernzerhof exchange correlation functional and calculations are performed using both standard DFT and the DFT+ *U* approach to correct for the self-interaction error. We employ the rotationally invariant DFT+ *U* formulation of Dudarev^44^ and the effective Hubbard parameter (*U*−*J*) for the Fe 3*d* states is taken as 3.8 eV as was employed in previous studies^37^,^45^. The valence-electron wavefunctions are expanded in a plane wave basis with energies up to 400 eV and structural optimization is performed until forces are <0.01 eV Å^−1^. For the bulk primitive cell of Fe_3_O_4_, a 7 × 7 × 7 Monkhorst–Pack *k*-point grid and similar *k*-point densities are employed in the supercell calculations. No symmetry constraints are employed in any of the structural optimizations reported and in general structures are optimized with respect to the positions of all ions and the length and orientation of all cell vectors. The only exception is for the APB calculations where the length of cell vectors parallel to the APB defect plane are fixed at the appropriate bulk values for the given method (that is, DFT or DFT+ *U*) and all angles are constrained to 90°. This is to ensure that the structures and formation energies obtained are appropriate for a semi-infinite bicrystal.

We note that DFT predicts the electronic ground state of Fe_3_O_4_ to be a half-metallic ferrimagnet, while DFT+ *U* predicts an insulating ferrimagnetic state due to polaronic charge ordering over octahedral iron sites. As discussed by Pinto and Elliott^37^, it is possible to obtain many different metastable electronic configurations using DFT+ *U* corresponding to different charge-ordering patterns consistent with the low transition temperature for the Verwey transition. With this method, we predict the lattice constant of the room temperature cubic phase of Fe_3_O_4_ to be 8.40 Å using DFT and 8.47 Å using DFT+ *U* in good agreement with the experiment.

The formation energy of the APB defects is defined as


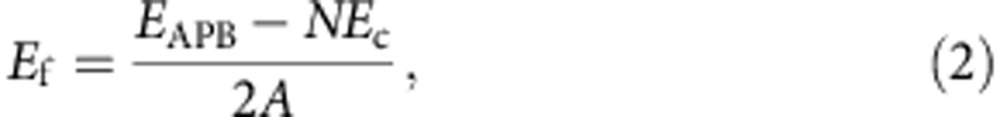


where *E*_APB_ is the energy of the supercell containing the two equivalent APB defects, *N* is the number of Fe_3_O_4_ formula units in the supercell, *E*_c_ is the calculated cohesive energy of Fe_3_O_4_ and *A* is the area of each APB defect in the supercell. The bulk cohesive energy is calculated using a cell of the same number of atoms and approximate dimensions as the defective supercell to minimize errors associated with differing *k*-point sampling and basis set quality.

### Material synthesis

Thin-foil samples of Fe_2_O_3_ with 
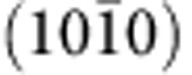
 exposed surfaces were prepared by cutting, mechanically grinding and dimpling commercial single-crystalline substrates down to 20 μm. In the final Ar ion-beam-thinning process, we applied accelerating gun voltages of 1.5–2.5 kV and incident beam angles of 4–6° to minimize radiation damage. After that, the thin-foil TEM specimens were annealed under an ambient pressure of 10^−4^ Pa at 973 K for 1 h. Following thermal annealing, the thin-foil TEM samples transformed into the Fe_3_O_4_ phase as revealed by the diffraction pattern and TEM and high-resolution TEM (HRTEM) observations, which also shows that the Fe_3_O_4_ phase has a good crystallinity.

We also examined a second type of sample obtained by deposition of Fe_3_O_4_ onto single-crystal MgAl_2_O_4_(111) substrates by molecular beam epitaxy methods^16^. Before deposition, a chamber base pressure of <2 × 10^−10^ mbar was achieved. Fe and atomic O were simultaneously deposited using a Knudsen cell and a radio frequency-assisted plasma source, respectively. Film growth has been performed at a nominal rate of 1.2 Å min^−1^, which has been corroborated by the close agreement of expected and actual (measured by cross-sectional TEM imaging) film thicknesses (±10%). During growth, the substrate was held at 350 °C in a partial pressure of 5 × 10^−6^ mbar of atomic oxygen supplied by the plasma source.

Film growth was monitored in real time using reflective high-energy electron diffraction (RHEED), with RHEED oscillations indicating layer by layer film growth. Chemical analysis was performed using *in situ* X-ray photoelectron spectroscopy to differentiate between competing iron-oxide phases and ensure film stoichiometry. Analysis of the Fe_3_O_4_/MgAl_2_O_4_(111) structure using selected area diffraction established the expected epitaxial relationship, Fe_3_O_4_(111)||MgAl_2_O_4_(111) and 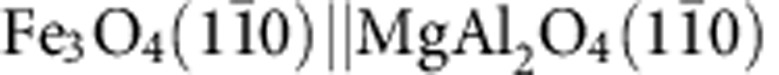. TEM analysis indicates an atomically sharp interface between film and substrate.

### Transmission electron microscopy

For the thin-foil samples, HAADF-STEM images were taken with a 200-kV STEM (JEM-ARM200F, JEOL) equipped with a probe corrector (CEOS, Gmbh), which offers an unprecedented opportunity to probe structures with sub-Ångström resolution. A probe current of 30 pA was used for the STEM imaging. The collection angle for the HAADF images was 90–175 mrad.

For the Fe_3_O_4_/MgAl_2_O_4_(111) sample, HRTEM, selected area diffraction and HAADF-STEM have been performed using a double-aberration-corrected field emission JEOL FS-2200 JEM TEM/STEM and a JEOL-2011 TEM (both operating at 200 kV). Cross-sectional microscope samples were produced by conventional methods, which include mechanical thinning and low-angle Ar ion milling to achieve electron transparency.

### TEM image simulation

HAADF-STEM image simulations have been performed using the multi-slice method implemented in QSTEM image simulation software^36^. Simulations are performed using experimentally determined parameters of the JEOL 2200 as determined by the CEOS aberration-correction software integral to the microscope and the design specifications of the microscope.

The electron beam parameters employed were: acceleration voltage =200 kV, chromatic aberration *C*_C_=1.6 mm, spherical aberration *C*_s_=0.0011, mm, fifth-order spherical aberration *C*_5_=1.756 mm, convergence semi-angle *α*=24 mrad and HAADF-STEM detector acceptance semi-angle 85–170 mrad. During microscope operation, the twofold astigmatism and focus are continuously corrected by the microscope user and can be identified manually from the live imaging and fast Fourier transforms of the live image. For these simulations, twofold astigmatism has been neglected.

The atomic coordinates used in this image simulation were taken from the relaxed coordinates of the DFT structural refinement calculations. By expressing the atomic coordinates in slab geometry and considering a number of sequential defect cells along the beam propagation direction, we produce a structural model of sufficient thickness (35.6 nm) such that these simulations represent the likely configuration of a real TEM specimen and reach a point where site intensity in the resultant simulation does not vary significantly from one slice to another. These simulations are performed over 240 projected potential slices of 1.4844 Å thickness, which ensures that the individual atomic planes of this crystal are all kept exclusively within a single computational slice.

The resultant image simulation is calculated over a 20 Å^2^ field of interest that includes a full repetition of the bulk Fe_3_O_4_ regions away from the structural {110} defect and the defect itself. This region is significantly over-sampled at 0.1 Å per pixel. The effect of thermal diffuse scattering has been modelled using a frozen phonon approximation assuming a room temperature (300 K) specimen. By implementing 30 thermal diffuse scattering iterations in these simulations, we have effectively modelled thermal scattering behaviour. These image simulations are completed by convolution with a Gaussian distribution to simulate the finite electron source size of the microscope and lens instabilities, and rescaling to a 1024 × 1024 pixel array^46^.

## Author contributions

K.P.M. led the theoretical part of the study and F.H. performed some of the calculations. D.G. and V.K.L. performed electron microscopy analysis of the the molecular beam epitaxy-grown Fe_3_O_4_ films and performed transmission electron microscopy image simulations. C.C. and Z.W. directed by Y.I. synthesized the thin-foil Fe_3_O_4_ samples and performed the transmission electron microscopy analysis. The manuscript was written by K.P.M. with input from all co-authors.

## Additional information

**How to cite this article**: McKenna, K. P. *et al*. Atomic-scale structure and properties of highly stable antiphase boundary defects in Fe_3_O_4_. *Nat. Commun.* 5:5740 doi: 10.1038/ncomms6740 (2014).

## Supplementary Material

Supplementary Information Supplementary Figures 1-4 and Supplementary Discussion.

Supplementary Dataset 1 Crystallographic Information File containing the supercell and coordinates for the (110) antiphase boundary defect (model APB-I) optimized using the PBE functional.

Supplementary Dataset 2 Crystallographic Information File containing the supercell and coordinates for the (110) antiphase boundary defect (model APB-I) optimized using the PBE+U functional.

Supplementary Dataset 3 Crystallographic Information File containing the supercell and coordinates for the (110) antiphase boundary defect (model APB-II) optimized using the PBE functional.

Supplementary Dataset 4 Crystallographic Information File containing the supercell and coordinates for the (110) antiphase boundary defect (model APB-II) optimized using the PBE+U functional.

Supplementary Dataset 5 Crystallographic Information File containing the supercell and coordinates for the Pmma phase of Fe3O4 optimized using the PBE functional.

Supplementary Dataset 6 Crystallographic Information File containing the supercell and coordinates for the Pmma phase of Fe3O4 optimized using the PBE+U functional.

## Figures and Tables

**Table 1:** Parameters of Buckingham interatomic potentials for Fe_3_O_4_.

**Species**		***A*** **(eV)**	***ρ*** **(Å)**	***C*** **(Å)**
Fe_tet_	O	1102.4	0.3299	0.00
Fe_oct_	O	812.0	0.3399	0.00
O	O	22,764.0	0.1490	15.0
