# Supplementary material for: Atomic-scale structure and properties of highly stable antiphase boundary defects in Fe3O4
Source: Nat Commun. 2014 Dec 10;5:5740. doi: 10.1038/ncomms6740 (PMC4275585; doi:10.1038/ncomms6740)
Supplement: Supplementary Information — Supplementary Figures 1-4 and Supplementary Discussion. [file ncomms6740-s1.pdf]

Supplementary Figures

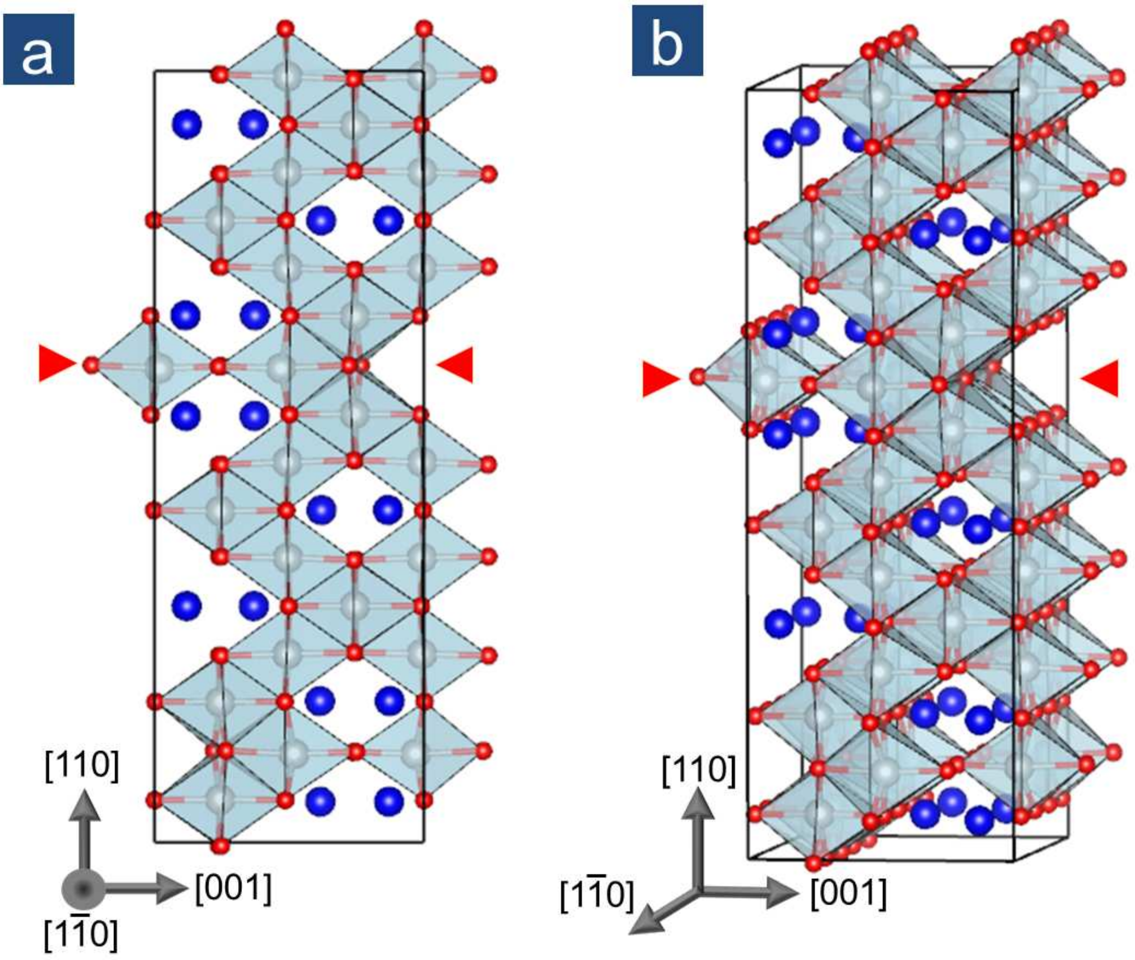

Supplementary Figure 1: **Predicted structure of the most stable {110} antiphase boundary defect in magnetite (model APB-I).** a) The same structure as that shown in Fig. 1b (main text) but with Fe octahedra highlighted. Small red spheres represent oxygen atoms, large dark blue spheres represent tetrahedral Fe atoms and gray spheres represent octahedral Fe atoms. Red arrows indicate the position of the APB defect. b) The same structure as shown in (a) but rotated to give a three-dimensional perspective.

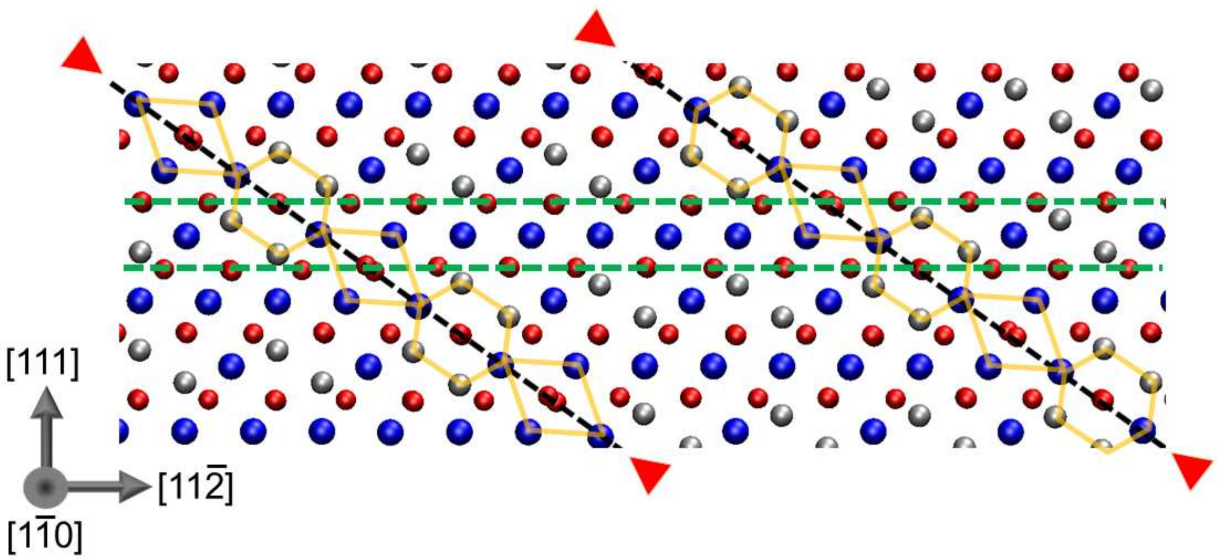

Supplementary Figure 2: **A different perspective on the APB-I defect.** The structure in Fig. 1b (main text) has been rotated so that the (111) oxygen planes run horizontally. The Fe sites in bulk magnetite sit between these oxygen planes in rows of either pure octahedral Fe or rows of mixed octahedral and tetrahedral Fe. The main effect of the APB is to introduce a switch between rows of pure octahedral Fe and rows of mixed octahedral and tetrahedral Fe (see row highlighted between the dashed green lines for an example). Small red spheres represent oxygen atoms, large dark blue spheres represent tetrahedral Fe atoms and light gray spheres represent octahedral Fe atoms. Red arrows indicate the position of the APB defects which run diagonally, orange diamonds and hexagons highlight the APB structural units.

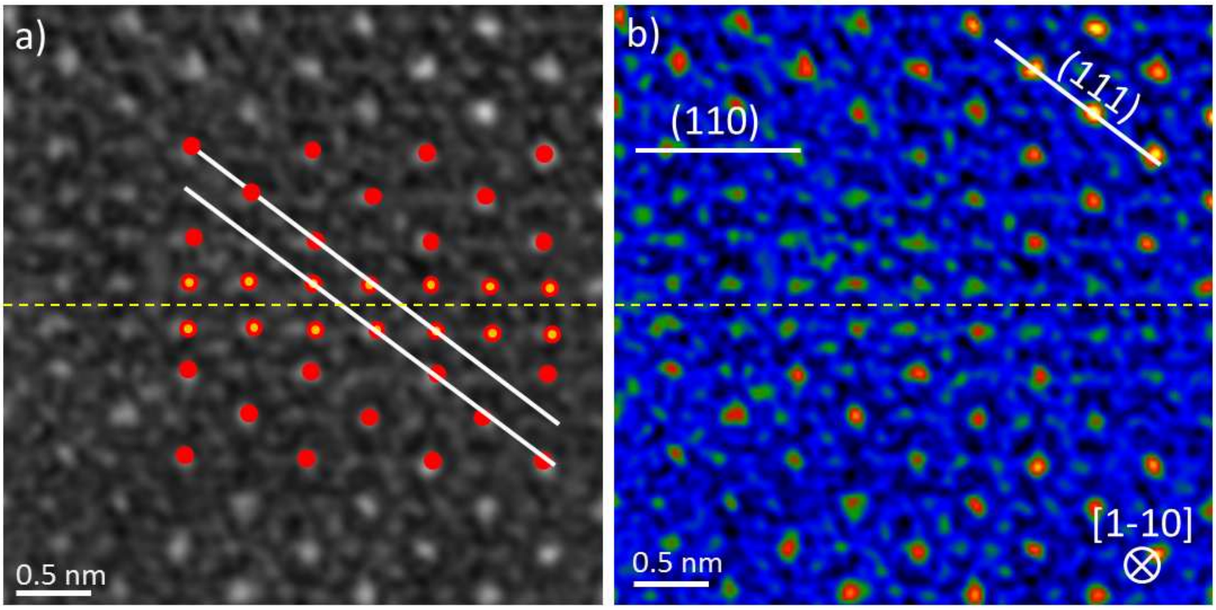

Supplementary Figure 3: **HAADF-STEM image of a (110) APB in the Fe_3O_4/$MgAl_2O_4$(111) sample imaged in the [1$\bar{1}$0] viewing direction.** The defect plane is highlighted with the dashed yellow line running horizontally across both image panes. (a) Shows the relative shift of the Fe_3O_4 structure at the APB. In this image the double occupied octahedral sites away from (at) the interface are identified with red circles (red and yellow circles) to show the discontinuity of the site occupation on the (111) planes (identified with white lines) across the boundary. (b) Shows the same image as (a) but rendered in a higher contrast full color view, this allows the specific sites identified in (a) to be seen more clearly. The defect which runs horizontally through these images propagates at an angle to the (111) growth plane of the film. The crystallographic geometry is identified in (b).

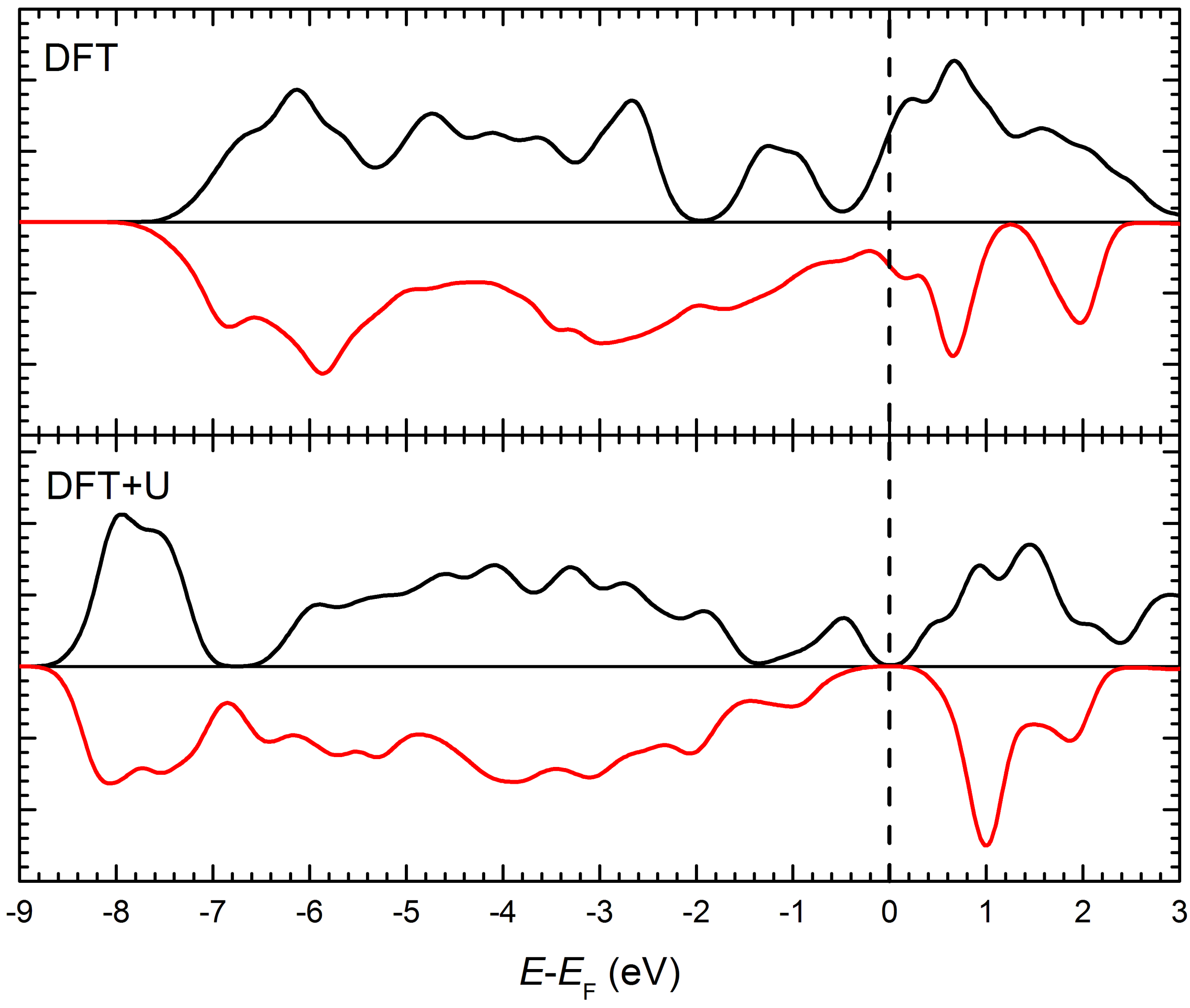

Supplementary Figure 4: **Electronic density of states of the Pmma phase.** a) At the DFT level the Pmma phase is predicted to be a half metallic with 35% spin polarization at the Fermi level. b) At the DFT+U level charge ordering over the octahedral Fe sites leads to the formation of an electrically insulating state.

Supplementary Discussion

Predicted structure of the $\{110\}$ APB The most stable $\{110\}$ APB (denoted APB-I) is characterized by a crystal translation $(1/4)a[110]$ and is shown in Fig. 1b in the main text. As an alternative we also show the structure in Supplementary Fig. 1 with Fe octahedra highlighted. Bulk Fe_3O_4 can be considered as a face centered cubic lattice of oxygen ions with octahedral and tetrahedral interstitial Fe sites. Supplementary Fig. 2 highlights the fact that in the bulk crystal Fe sites sit between (111) oxygen planes in rows of either pure octahedral Fe or rows of mixed octahedral and tetrahedral Fe sites. At the APBs there is a switch between rows of pure octahedral Fe to rows of mixed octahedral and tetrahedral Fe (and vice versa). However, the overall number of octahedral and tetrahedral Fe sites is unchanged and the structural distortion is small helping to explain the very low formation energy that is calculated.

$\{110\}$ APB in Fe_3O_4/$MgAl_2O_4$(111) sample Supplementary Fig. 3 shows a HAADF-STEM image of a $\{110\}$ APB in the Fe_3O_4/$MgAl_2O_4$(111) sample imaged in the $[1\bar{1}0]$ viewing direction. Clear evidence of an APB can be seen in Supplementary Fig. 3a where the position of the highly occupied Fe sites (red spheres) can be seen to shift out of sequence on the (111) planes (white solid line) across the defect boundary (yellow dashed line). By studying both (a) and (b) it can be seen that the indicative hexagon-diamond-hexagon structure of the refined structure is missing in these images, instead we observe twice as many twinned bright sites as we would expect from the structural model. The results we observe are consistent with two overlapping grains of the refined structure viewed in projection such that the double sites are observed in two unique locations;

this would lead to an overlaying of the hexagon-diamond-hexagon motif observed in the structural models and the annealed Fe_2O_3 samples and create the extra sites we observe in Supplementary Fig. 3. As these TEM specimens have been grown and prepared in very different conditions than the Fe_2O_3 annealing method it is reasonable to assume that some granular growth characteristics may remain within the film as a result of 3D growth effects. Furthermore, the greater physical thickness of these MBE grown TEM specimens, perhaps $>$80 nm makes it likely we would image such behavior.

Supplementary Fig. 3b shows the crystallographic planes of this film. The image is rotated to present the defect horizontally, however the growth has been propagated in the (111) direction which can be seen at an angle running through a line of octahedral Fe sites to the top right of the image.

Predicted electronic properties of the Fe_3O_4 Pmma phase At the DFT level the Pmma phase is predicted to be an ferrimagnetic material with a magnetic moment of 4.17 μ_B per formula unit ($a = 2.961$, $b = 8.569$, $c = 5.943$). The phase is predicted to the 606 meV per formula unit less stable that the bulk cubic phase and the electronic ground state is predicted to be metallic with a 35% spin polarization at the Fermi level. At the DFT+U level the Pmma phase is also predicted to be a ferrimagnetic material with a magnetic moment of 3.90 μ_B per formula unit ($a = 3.086$ Å , $b = 8.654$ Å and $c = 5.898$ Å). The electronic ground state is predicted to be insulating as a result of charge ordering over the octahedral Fe sites. Supplementary Fig. 4 shows the density of states calculated at both the DFT and DFT+U levels.

Structural information available online Crystallographic Information Files containing the optimized structures of APB-I, APB-II and the Pmma phase at the DFT and DFT+U levels are available online.
